# UK Fixation of Distal Tibia Fractures (UK FixDT): protocol for a randomised controlled trial of ‘locking’ plate fixation versus intramedullary nail fixation in the treatment of adult patients with a displaced fracture of the distal tibia

**DOI:** 10.1136/bmjopen-2015-009162

**Published:** 2015-09-16

**Authors:** Juul Achten, Nicholas R Parsons, Katie R McGuinness, Stavros Petrou, Sarah E Lamb, Matthew L Costa

**Affiliations:** 1 Warwick Medical School, The University of Warwick, Coventry, UK; 2 Nuffield Department of Orthopaedics, Rheumatology & Musculoskeletal Sciences, University of Oxford, Oxford, UK

**Keywords:** ACCIDENT & EMERGENCY MEDICINE, HEALTH ECONOMICS, ORTHOPAEDIC & TRAUMA SURGERY

## Abstract

**Introduction:**

The treatment of displaced, extra-articular fractures of the distal tibia remains controversial. These injuries are difficult to manage due to limited soft tissue cover, poor vascularity of the area and proximity to the ankle joint. Surgical treatment options are expanding and include locked intramedullary nails, plate and screw fixation and external fixator systems. The nail and plate options are most commonly used in the UK, but controversy exists over which treatment is most clinically and cost-effective. In this multicentre randomised controlled trial we aim to assess ratings of disability 6 months postinjury in patients who have sustained a distal tibia fracture treated with either an intramedullary nail or plate and locking screw fixation.

**Methods and analysis:**

Adult patients presenting at trial centres with an acute fracture of the distal tibia will be considered for inclusion. A total of 320 patients will provide 90% power to detect a difference of 8 points in Disability Rating Index (DRI) score at 6 months at the 5% level. The randomisation sequence is stratified by trial centre and age, and administered via web-based service with 1:1 treatment allocation. Baseline demographic and pre-injury functional data and radiographs will be collected using the DRI, Olerud and Molander, and EuroQol EQ-5D questionnaire. Clinical assessment, early complications and radiographs will be recorded at 6–8 weeks. Functional outcome, health-related quality of life and resource use will be collected at 3, 6 and 12 months postoperatively. The main analysis will investigate differences in DRI 6 months postsurgery, between the two treatment groups, on an intention-to-treat basis. Tests will be two-sided and considered to provide evidence for a significant difference if p values are <0.05.

**Ethics and dissemination:**

NRES Committee West-Midlands, 6/11/2012 (ref:12/WM/0340). The results of the trial will be disseminated via peer-reviewed publications and presentations at relevant conferences.

**Trial registration number:**

ISRCTN99771224.

**Strengths and limitations of this study**

- This will be the first multicentre randomised trial to assess the clinical and cost-effectiveness of intramedullary nail versus plate and locking screw fixation for patients with an extra-articular fracture of the distal tibia.
- Methodological qualities of the trial include: large number of intervention sites, optimised protocol to reduce risk of bias, appropriate sample size calculation and planned intention-to-treat analysis.
- The challenge for this study will be a potential lack of equipoise among trauma surgeons with regard to the treatment of this complicated type of fracture.

## Background

The tibia is the most commonly broken major bone in the leg. Injuries usually require hospital admission and frequently require surgery, resulting in prolonged periods (months) away from work and social activities.

The treatment of displaced, extra-articular fractures of the distal tibia (lower third) remains controversial. These injuries are difficult to manage due to the limited soft tissue cover, poor vascularity of the area and proximity of the fracture to the ankle joint. Infections, non-union and mal-union, are well-recognised complications.

Non-operative treatment is one option and avoids the risks associated with surgery. Sarmiento *et al*,^1^ in 2003, reviewed 450 closed fractures of the distal tibial following functional bracing: 13.1% developed a mal-union (defined as >7° of angulation or 12 mm shortening). A further study using a more robust definition of 10 mm shortening and 5° angulation found a higher rate of mal-union (26.4%).^2^ Bostman *et al*^2^ treated patients using a long leg cast and failure to maintain reduction led to surgical treatment with an intramedullary nail. Thirty-two of 103 cases required nailing at a mean of 9 days following injury. Two patients in this group and three in the non-operative group went on to have a non-union.^2^ Union rates were faster with intramedullary nailing compared with conservative treatment—median values were 12.5 and 14.5 weeks, respectively (p<0.001).^2^ Digby *et al*^3^ also found that non-operative treatment for tibial fractures in the metaphyseal region leads to unacceptable deformity and ankle stiffness. Therefore, non-operative treatment is not the treatment of choice in the majority of patients with a fracture of the distal tibia.

Surgical treatment options are expanding and include locked intramedullary nails, plate and screw fixation, as well as external fixator systems including the Ilizarov frame and hybrid fixators. External fixators may be beneficial in selected cases—particularly those with severe soft tissue injuries—but, in the UK, the nail and plate options are most commonly used for extra-articular fractures. Mid-shaft fractures of the tibia are generally successfully treated with locked intramedullary nails. However, in the more distal metaphyseal region of the tibia, the fixation may be less stable.^4^ The nail or screws that are inserted into the nail may break,^5^ mal-alignment may occur,^6^ and there is a risk that the nail will penetrate into the ankle joint.^7^ ^8^

The development of ‘locking’ plates (where a thread on the head of the screws locks into the holes in the plate to create a ‘fixed-angle’ construct) has led to a recent increase in the use of plate fixation. However, plates are not without risks, they require greater soft tissue dissection, which carries a risk of infection, wound breakdown and devitalisation of the surrounding tissue.^9^

In a retrospective study of 111 patients with extra-articular fractures of the distal tibia (4–11 cm proximal to the plafond), a comparison was made between intramedullary nailing and plate fixation. Seventy-six fractures were treated with an intramedullary nail and 37 were treated with a medial plate.^10^ Nine patients (12%) had a delayed union or non-union in the intramedullary nail group and one patient (2.7%) had a non-union after plate fixation (p=0.10). Angular mal-alignment of ≥5° occurred in 22 patients with nails (29%) and 2 with plates (5.4%, p=0.003). The authors concluded that fractures of the distal tibia may be treated successfully with plates or nails, but that delayed union, mal-union and secondary procedures were more frequent after intramedullary nailing. Janssen *et al*^11^ found similar results, delayed union was higher in the intramedullary nail group (25%) compared to the plate fixation group (16.7%), rotational mal-alignment was also higher in the intramedullary nailing group (16.7%) compared with 0% in the plate group. However, this was not a randomised controlled trial (RCT) and the results do need to be interpreted with some caution. Randomised prospective assessment will be necessary to further clarify these issues and provide information about costs associated with these fractures.^10^

Only two prospective RCTs have been published to date. In the first, 64 patients were randomised either to intramedullary nail or plate fixation, for the treatment of a closed extra-articular fracture.^12^ The time to union was found to be similar for the two groups, and there was no difference in terms of Olerud and Molander Ankle Score (OMAS) at 2 years. However, a significant difference was observed in the number of wound complications: one in the intramedullary nail group versus seven in the plate group. This paper concludes that intramedullary nailing is the treatment of choice for this injury. However, the method of randomisation was poorly described and so bias in group assignment may have occurred. The study used traditional (non-‘locking’ plates) rather than the newer fixed-angle devices. Furthermore, the study included patients with Tschene classification C2 soft tissue injuries, which may have influenced the results. The second trial randomised 111 patients either to intermedullary nail fixation or ‘locking’ plate fixation.^13^ This trial also showed no difference in the time to union but, 1 year after the injury, there was some evidence of improved American Orthopaedic Foot and Ankle Society functional scores in the nail group. However, this was a single-centre investigation and over 20% of the patients in the trial were lost to follow-up.

In a meta-analysis, Zelle *et al*^14^ reviewed 1125 extra-articular fractures of the distal tibia. They reported that non-union, mal-union and infection rates were similar for patients undergoing intramedullary nailing and plate fixation. It must be noted that none of the studies in the review were RCTs.

## Pre-pilot data

We performed a pilot study involving 24 patients with extra-articular fractures of the distal tibia that were closed, or Gustilo and Anderson grade 1.^15^ The study was a RCT with clinical assessment, functional outcomes and radiological images performed at baseline, 6 weeks, and 3, 6 and 12 months postsurgery. The study was performed to obtain an estimate of the potential effect size to inform the sample size calculation for a larger definitive trial, and to assess recruitment rates and study feasibility.

The study had 12 patients in each group. There was no statistically significant difference between the groups 6 months after the injury but there was a 10-point difference (SD 20) in the Disability Rating Index (DRI)^16^ in favour of the intramedullary nail group. More secondary procedures were required in the ‘locking’ plate fixation group. There was also a large difference in the cost of the implants.

This pilot study provides compelling evidence to support the development of a definitive RCT in multiple centres.

## Trial design

The trial will be carried out in accordance with Medical Research Council Good Clinical Practice^17^ and applicable UK legislation, using the following protocol. The trial will be reported in line with the CONSORT statement.^18^

## Null hypothesis

There is no difference in the DRI at 6 months after injury between adults with a displaced fracture of the distal tibia treated with ‘locking’ plate fixation versus intramedullary nail fixation.

## Objectives

The primary objective is:

-  To quantify and draw inferences on observed differences in the DRI between the trial treatment groups at 6 months after injury.

The secondary objectives are:

1. To quantify and draw inferences on observed differences in early functional status at 3 months and later functional status at 12 months.
2. To quantify and draw inferences on observed differences in the radiological outcomes: non-union, mal-alignment and shortening.
3. To identify any differences in health-related quality of life between the trial treatment groups in the first year after injury.
4. To determine the complication rate of intramedullary nail fixation versus ‘locking’ plate fixation in the first year after injury.
5. To investigate, using appropriate statistical and economic analytical methods, the resource-use, costs and comparative cost-effectiveness of intramedullary nail fixation versus ‘locking’ plate fixation.

## Trial summary

The proposed project is a two-phased study. Phase 1 (internal pilot) will determine the expected rate of recruitment in a large-scale multicentre RCT in this complicated area of trauma research. Phase 2 (main phase) will be the proposed RCT in a minimum of 18 trauma centres across the UK.

### Internal pilot summary

The pilot will take place in six centres over a period of 6 months. The main aim of this initial phase will be to determine the number of eligible and recruited patients in the trauma centres over the course of 6 months. Screening logs will be kept at each site to determine the number of patients assessed for eligibility and reasons for any exclusion. In addition, the number of eligible and recruited patients, and the number of patients who decline consent/withdraw, will be recorded.

#### Main RCT summary

All adult patients presenting at the trial centres with an acute fracture of the distal tibia are potentially eligible to take part in the trial. The broad eligibility criteria will ensure that the results of the study can readily be generalised to the wider patient population. A computer-generated randomisation sequence, stratified by centre and age, will be produced and administered independently by a secure web-based service. Randomisation will be on a 1:1 basis to either intramedullary nailing or ‘locking’ plate fixation. Both of these operations are widely used within the NHS and all of the surgeons in the chosen centres will be familiar with both techniques.

Baseline demographic data, radiographs and pre-injury functional data using the DRI and the OMAS Questionnaire will be collected. The patients will also be asked to fill out the EuroQol EQ-5D health-related quality-of-life questionnaire twice at baseline; once to indicate their typical preinjury health status and a second time to indicate their current ‘postinjury’ status.

A research associate will perform a clinical assessment and record any early complications at 6 weeks, and a radiograph will be taken. A further clinical assessment and radiograph will also be taken at 12 months postoperatively to detect late complications. Functional outcome, health-related quality of life and resource-use questionnaires will be collected at 3 months, 6 months and 12 months postoperatively.

## Outcome measures

The primary outcome measure for this study is the *DRI*.^16^ The DRI score is a validated questionnaire that is self-reported (filled out by the patient). It consists of 12 items specifically related to function of the lower limb. This data will be collected at baseline, 3, 6 and 12 months postoperatively (table 1). The DRI has been proven to be a robust and practical clinical and research instrument, with good responsiveness and acceptability for assessment of disability caused by impairment in the lower limb.

**Table 1:** Follow-up measures

Time point data collection	
Baseline	DRI, OMAS, EQ-5D *preinjury*, *EQ-5D current* and radiographs
6 weeks	Complication records, radiographs and operative record
3 months	DRI, OMAS, EQ-5D, record of complications/rehabilitation or other interventions and resource-use questionnaire
6 months	DRI, OMAS, EQ-5D, record of complications/rehabilitation or other interventions and resource-use questionnaire
12 months	DRI, OMAS, EQ-5D, radiographs, record of complications/rehabilitation or other interventions and resource-use questionnaire

DRI, Disability Rating Index; OMAS, Olerud and Molander Ankle Score.

The secondary outcome measures in this trial:

*OMAS* is a self-administered patient questionnaire. It is a good outcome tool for assessing symptoms after an ankle fracture. The score is based on nine different items: pain, stiffness, swelling, stair climbing, running, jumping, squatting, supports and work/activities of daily living.^19^ The scoring system correlates well with parameters considered to summarise the results after this type of injury and is therefore recommended for use in scientific investigations.

*EQ-5D* is a validated, generic health-related quality-of-life measure consisting of five dimensions, each with a 3-level answer possibility. Each combination of answers can be converted into a health utility score.^20^ It has good test–retest reliability, is simple for patients to use and gives a single preference-based index value for health status that can be used for broader cost-effectiveness comparative purposes.

*Complications*: All complications will be recorded, including mal-union, delayed/non-union, infection, wound complications, vascular and neurological injury, and venous thromboembolism. A record will also be kept of any other surgery required in relation to the index fracture, including removal of any metalwork.

*Radiographic evaluation*: Standard anteroposterior and lateral radiographs of the tibia and fibula will be taken at baseline, 6 weeks and 12 months after the injury. If a radiograph is not taken at 12 months, say for example, the fracture has been considered by the treating surgeon to be healed at an earlier time point, the last radiograph taken before 12 months will be collected. These are radiographs routinely used for the investigation of patients with a suspected fracture of the distal tibia and for the follow-up of such patients following any intervention, so there will be no need to request any additional or special investigations.

We will use techniques common in long-term cohort studies to ensure minimum loss to follow-up, such as collection of multiple contact addresses and telephone numbers, mobile telephone numbers and email addresses. Considerable efforts will be made by the trial team to keep in touch with patients throughout the trial by means of newsletters, etc.

## Sample size

The minimum clinically important difference (MCID) is eight points on the primary outcome (DRI) measurement scale. The DRI is a 12-question, patient reported, functional outcome measure (physical exercise or sports, running, heavy physical work, heavy lifting, carrying a bag, leaning over a wash-stand, making a bed, moderate physical work, walks, mounting stairs, sitting still more than briefly and dressing or undressing) converted to a 100-point scale where ‘0’ represents normal function and ‘100’ represents complete disability. At an individual patient level, a difference of eight points represents the ability to climb stairs or run, with ‘some difficulty’ versus with ‘great difficulty’. At a population level, eight points represents the difference between a ‘healthy patient’ and a ‘patient with a minor disability’. Eight points also corresponds approximately to the clinically worthwhile benefit identified in other studies and the difference between treatment group means in our pilot study.

The SD of the DRI in our pilot study was approximately 20 points; the sample size has also been estimated for a larger and smaller SD to obtain an indication of the sensitivity to changes in this parameter. Assuming the distribution of DRI in the study populations to be approximately normal, which is consistent with assumptions made for other reported trials using DRI as the primary outcome measure, table 2 shows the total trial sample size with two-sided significance set at 5% for various scenarios of power and sample SD.

**Table 2:** Sample size at variable power and SD

	Power	
SD	80%	90%
15	112	150
20	198	**264**
25	308	412

Bold typeface indicates the actual sample size chosen for this trial.

The bold figure of 264 patients represents the most likely scenario, based on our current knowledge, for 90% power to detect the selected MCID. Allowing a margin of 20% loss during follow-up, this gives a figure of *320 patients in total*. Therefore, 160 patients randomised to each group will provide 90% power to detect a difference of eight points in DRI at 6 months with 90% power at the 5% level.

### Eligibility

Patients will be eligible for this study if:

- Aged 16 years or over;

Patients with a fracture that involves the distal tibial metaphysis—defined as a fracture extending within 2 Müller squares of the ankle joint^21^—are eligible if:

- The fracture is closed;
- In the opinion of the attending surgeon, the patient would benefit from internal fixation of the fracture.

Patients will be excluded from participation in this study if:

- In the opinion of the attending surgeon, there is a contraindication to intramedullary nailing;
- The fracture is open;
- There is a contra-indication to anaesthesia;
- There is evidence that the patient would be unable to adhere to trial procedures or complete questionnaires
- The fracture extends into the ankle joint (ie, intra-articular fracture).

Contraindications to intramedullary nailing are: the medullary canal is too narrow OR there is a preinjury deformity of the medullary canal OR it is not possible to achieve fixation of four cortices with screws distal to the fracture. We feel that these exclusion criteria will be easily understood by the surgeons and are in keeping with the pragmatic nature of the trial. However, we will include the specific reason in the trial screening data. For those patients withdrawing from the trial after written consent has been obtained, data obtained up until the point of withdrawal will be included in the final analysis.

### Recruitment

The internal pilot will specifically inform and test the recruitment rate for the main trial. Recruitment will take place in six trial centres over a period of 6 months. The expected rate of recruitment is based on a pre-pilot study performed at the lead centre. The average recruitment rate for this pre-pilot study, during which 24 patients were recruited, was 1.3 patients per month. The other centres involved in the trial will all be regional trauma units with similar catchment areas to the lead centre. Experience from previous multicentre trials has, however, shown that recruitment outside of the lead centre tends to occur at a lower rate. As such, a conservative recruitment rate of 0.75 patient per month per centre is estimated for this trial. If this recruitment rate can be achieved by the end of the internal pilot, the trial will progress to the main phase. We intend to recruit patients from a minimum of 18 centres in total (including the lead centre). The sample of 320 patients will be recruited over a 30-month period.

Screening logs will be collected throughout the trial to assess the main reasons for patient exclusion as well as the number of patients unwilling to take part. Patients will be screened by the Research Associates in the Emergency Department and Fracture Clinics at the trial centres. Any patient over 16 years of age, with a fracture of the distal tibia who, in the opinion of the treating surgeon, would benefit from internal fixation, will potentially be eligible for the trial. The trial will act in accordance with the Mental Capacity Act 2005 and the procedures for undertaking trials in ‘emergency settings’ will be followed as described in detail below in section 3.6.3 (consent) of this protocol. The consent procedures will be reviewed at the end of the pilot period.

### Consent

The clinical team responsible for patient care will make the decision regarding patient capacity. Informed consent from the patient will be obtained by the local research associate. Patients will be provided with verbal and written information about the study. In general, patients who are admitted with a fracture of the distal tibia will have their surgery on the next available trauma list. Timing and appropriateness of obtaining consent in this setting will be closely monitored during the internal pilot, and reviewed by the independent Trial Steering Committee.

For those patients withdrawing from the trial after written consent has been obtained, data obtained up until the point of withdrawal will be included in the final analysis.

Any new information that arises during the trial that may affect participants’ willingness to take part will be reviewed by the Trial Steering Committee; if necessary, this will be communicated to all participants. A revised consent form will be completed if necessary.

### Randomisation

The method of fixation will be allocated using a secure, centralised, web-based randomisation service. The randomisation service will be available 24 h each day to facilitate the inclusion of all eligible patients. The allocated treatment will then be reported to the research associate who will inform the treating surgeon. The surgeon will then arrange the allocated surgery on the next available trauma operating list, as per standard practice at that institution; this will ensure the integrity of the randomisation process. Randomisation will be implemented using a minimisation algorithm (sometimes referred to as *adaptive randomisation*) that attempts, at recruitment of each new patient, to balance the marginal totals for each level of the stratification factors identified below. This is the usual practice for trials run at Warwick clinical trials unit (CTU). Experience indicates that, for studies where some centres recruit only a relative small number of patients, this method tends to perform better than conventional stratification methods.

*Stratification by centre* will help to ensure that any clustering effect related to the centre itself will be equally distributed in the trial arms. The catchment area (the local population served by the hospital) will be similar for all of the hospitals, each hospital being a trauma unit dealing with these fractures on a daily basis. While it is possible that the surgeons at one centre may be more expert in one or the other treatment than those at another centre, all of the recruiting hospitals have been/will be chosen on the basis that both techniques are currently routinely available at the centre, that is, theatre staff and surgeons will already be equally familiar with both forms of fixation. This cannot eliminate the surgeon-specific effect of an individual at any one centre.^22^ However, fixation of a fracture of the tibia is not an uncommon procedure and many surgeons will be involved in the management of this group of patients: between 10 and 30 surgeons at each centre, including Consultants and Trainees. Therefore, we anticipate that each individual surgeon will only operate on 2–3 patients enrolled in the trial, greatly reducing the risk of a surgeon-specific effect on the outcome at any one centre.

*Stratification on the basis of age* will be used to discriminate between younger patients with normal bone quality sustaining high-energy fractures, and older patients with low-energy (fragility) fractures related to osteoporosis. The stratification will help to identify any effect related to the quality of the patients’ bone. The use of DEXA (dual-energy X-ray absorptiometry) is widely regarded as the gold standard for the assessment of bone density. However, such an investigation may be expensive and not routinely available at all centres.

Therefore, we propose to use age as a surrogate for bone density. In a large study in Norway, involving 7600 participants, it was demonstrated that bone mineral density remains stable up until the age of 50 years. After the age of 50 years, bone mineral density decreased steadily in males, while in females there was an initial decline between the ages of 50 and 65 years, with a further decline in both age groups thereafter.^23^ Over 1000 patients with a fracture were recently assessed in a study by Court-Brown and Caeser.^24^ This study confirmed that there is a clear bimodal distribution according to the age of the patient. The crossover of the two peaks of incidence was around 50 years of age. These studies provide strong evidence that patients over 50 years of age become increasingly vulnerable to fragility fractures. Therefore, we have chosen an age of 50 years as the stratification cut-off for this trial.

### Postrandomisation withdrawals

Participants may decline to continue to take part in the trial at any time and without prejudice. A decision to decline consent or withdraw will not affect the standard of care the patient receives.

Participants have two options for withdrawal:

1. Participants may withdraw from completing any further questionnaires but allow the trial team to continue to anonymously view and record any relevant hospital data that is recorded as part of normal standard of care; including X-rays and further surgery information.
2. Or participants can withdrawal wholly from the study and only data obtained up until the point of withdrawal will be included in the final analysis of the study; thereafter no further data will be collected for that participant.

Once withdrawn, the patient will be advised to discuss their further care plan with their surgeon.

### Blinding

As the type of fixation used requires clearly visible surgical scars, the patients cannot be blind to their treatment. In addition, the treating surgeons will also not be blind to the treatment, but will take no part in the postoperative assessment of the patients. The functional outcome data will be collected and entered into the trial central database via questionnaire by a research assistant/data clerk in the trial central office. The X-rays collected will be reviewed by an independent assessor.

## Trial treatments

All the hospitals involved in this trial currently use both methods of fixation and all the consulting surgeons involved will be familiar with both techniques. Operative fixation of fractures of the distal tibia usually takes place under a general anaesthetic, but this decision will be made by the attending anaesthetist.

Each patient will undergo the allocated surgery according to the preferred technique of the operating surgeon. However, the basic principles of intramedullary nailing and ‘locking’ plate fixation are inherent in the technique (see below), there are several different implant systems and several different options for the positioning of the screws. Similarly, each surgeon will make minor modifications to their surgical technique according to preference and the specific pattern of each fracture. In this trial, the details of the surgery will be left entirely to the discretion of the surgeon, to ensure that the results of the trial can be generalised to as wide a group of patients as possible. However, a copy of the ‘operating record’ will form part of the trial data set.

Although all of the surgeons in the trial will be familiar with both techniques, it is possible that an individual surgeon may have more experience with one technique than the other. We expect that the proficiency of an individual surgeon to perform the procedure may change over time, as the surgeon gains experience and expertise. The term ‘learning curve’ is often used to describe this process. It will be important to monitor the learning curve for all surgeons throughout the trial. The operating time recorded on the operative record for each surgery will be used as a proxy to measure the task efficiency of the surgeons (quality assurance of the clinical process), and the number of complications (eg, infections) at 6 weeks after surgery will also be recorded as a patient-based outcome. Given the number of centres and surgeons taking part in this trial, no individual surgeon will perform more than a small number of the procedures. However, where data are available for individual surgeons, temporal variations in operation times and complications at 1 week will be modelled for each surgeon using a power curve for the trend, with appropriate adjustment for confounding factors such as the age of the patients.^22^ Also, as this study involves multiple surgeons, in addition to multiple centres, we anticipate that a more complex hierarchical model^16^ that fully accounts for the structure of the data may prove to be useful; we therefore anticipate fitting models of this type in the analysis. Results from the learning curve analysis for each surgeon will inform inferences regarding overall treatment differences and, if necessary, guide recommendations for implementation and training.

### Intramedullary nailing

The intramedullary nail is inserted at the proximal end of the tibia and passed down the centre (medullary canal) of the bone in order to hold the fracture in the correct (anatomical) position. The reduction technique, the surgical approach, the type and size of the nail, the configuration of the proximal and distal interlocking screws, and any supplementary device or technique, will be left entirely to the discretion of the surgeon as per standard clinical practice.

### ‘Locking’ plate fixation

The ‘locking’ plate is inserted at the distal end of the tibia and passed under the skin onto the surface of the bone. Again, the details of the reduction technique, the surgical approach, the type and position of the plate, the number and configuration of fixed-angle screws and any supplementary device or technique, will be left to the discretion of the surgeon. The only stipulation is that fixed-angle screws must be used in at least some of the distal screw holes—this is standard practice with all distal tibia ‘locking’ plates.

### Rehabilitation

We will ensure that all patients randomised into the two groups will receive the same standardised, written physiotherapy advice detailing the exercises they need to perform for rehabilitation following their injury. All of the patients in both groups will be advised to move their toes, ankle and knee joints fully within the limits of their comfort. Weight-bearing status will be decided by the treating surgeon. In this pragmatic trial, any other rehabilitation input beyond the written physiotherapy advice (including a formal referral to physiotherapy) will be left to the discretion of the treating clinicians. However, a record of any additional rehabilitation input (type of input and number of additional appointments) together with a record of any other investigations/interventions will be requested as part of the 3-month, 6-month and 12-month follow-ups, and this will also form part of the trial data set.

### Follow-up

Baseline, standardised radiographs will be copied from the hospital picture archiving and communication (PAC) system. Copies of the baseline clinical report forms (CRFs) and images will be delivered to the trial co-ordinating centre.

As part of routine clinical practice, patients will be seen in clinic on a regular basis after this injury. Any further clinical follow-up in the first year after the injury will be at the discretion of the surgeon, but will not influence the collection of the standard outcome data. For this trial, the primary outcome point will be at 6 months, when patients with an uncomplicated fracture may expect to return to normal activities; but to ensure that all complications and secondary procedures are captured, we propose to continue follow-up for 1 year.^13^

The research associate will perform a clinical assessment and make a record of any early complications at 6 weeks. Radiographs will be taken at 6 weeks and 12 months. The radiograph at 6 weeks will be used to assess the quality of the reduction: mal-alignment will be defined as more than 10 mm of shortening and more than 5° of angulation in any plane.^2^ An uncomplicated fracture of the distal tibia would be expected to be clinically united at 6 months after the injury. The primary functional outcome measure will therefore be collected at 6 months. However, radiographic union may lag behind the clinical picture. Therefore, the 12-month radiographs will be used as the definitive radiographic assessment of alignment^2^ and to assess if there are long-term complications, including non-union (failure to show bridging callus on three of four cortices on orthogonal radiographs) and arthritis of the ankle joint (joint space narrowing with osteophyte formation and peri-articular sclerosis).

The functional outcome data will be collected using questionnaires at 3, 6 and 12 months postoperatively. In addition to the DRI, the patients will be asked to fill out the EuroQol questionnaire, a complications/further surgical interventions and resource-use questionnaire. Patients will be asked to complete their 6-month and 12-month postoperative questionnaire during their routine follow-up appointments. The 3-month postoperative questionnaires, short annual questionnaires and any ‘missed’ questionnaires, will be sent to the patients through the post; a process carried out centrally by a data clerk at the Warwick Clinical Trials Unit. All the outcome questionnaires can be completed over the phone if postal copies are not returned. Text messages may be sent to patients to inform them that a questionnaire is due or on its way. Text messages will only be sent to those patients who have given their prior consent to this by initialling the corresponding box on the consent form. Text messages will be sent via the Warwick Clinical Trials Unit mobile phone from a secure office. The lead site (University of Warwick) will request a copy of the consent form from each patient entered into the study to determine if the patient has consented to text messages before a message is sent out.

Thereafter, patients who have consented to the ‘long-term’ follow-up will be sent an annual postal questionnaire for ongoing surveillance.

## Adverse event management

Adverse events (AEs) are defined as *any untoward medical occurrences in a clinical trial subject that do not necessarily have a causal relationship with the treatment*. All AEs will be listed on the appropriate Case Report Form for routine return to the ‘FIXDT’ central office.

Serious adverse events (SAEs) are defined as any *untoward and unexpected medical occurrence that:*

1. Results in death;
2. Is life-threatening;
3. Requires hospitalisation or prolongation of existing inpatients’ hospitalisation;
4. Results in persistent or significant disability or incapacity;
5. Is a congenital anomaly or birth defect;
6. Is any other important medical condition that, although not included in the above, may require medical or surgical intervention to prevent one of the outcomes listed.

All SAE will be entered onto the Serious Adverse Event reporting form and faxed to a dedicated fax machine at Warwick Clinical Trials Unit within 24 h of the investigator becoming aware of them. Once received, causality and expectedness will be confirmed by the Chief Investigator. SAEs that are deemed to be unexpected and related to the trial will be notified to the Research Ethics Committee (REC) and sponsor within 15 days. All such events will be reported to the Trial Steering Committee and Data Monitoring Committee at their next meetings.

SAEs that may be expected as part of the surgical interventions, and that do not need to be reported to the main REC, are: complications of anaesthesia or surgery (eg, wound complications, infection, damage to a nerve or blood vessel and thromboembolic events) and secondary operations for or to prevent infection, mal-union, non-union or for symptoms related to the metalwork. All participants experiencing SAEs will be followed-up until the end of the trial, per protocol.

### Risks and benefits

The risks associated with this study are predominantly those associated with the surgery: infection, bleeding and damage to the adjacent structures such as nerves, blood vessels and tendons. Participants in both groups will undergo surgery and will potentially be at risk from any/all of these complications. There are no data to suggest that the risk is greater in one or the other group. We believe that the overall risk profile is similar for the two interventions but assessment of the number of complications in each group is a secondary objective of this trial.

## Data management

The Case Report Forms will be designed by the trial coordinator in conjunction with the trial management team. All electronic patient-identifiable information will be held on a secure, password-protected database accessible only to essential personnel. Paper forms with patient-identifiable information will be held in secure, locked filing cabinets within a restricted area of Warwick Medical School. Patients will be identified by a code number only. Direct access to source data/documents will be required for trial-related monitoring. All paper and electronic data will be retained for at least 5 years after completion of the trial.

## Statistical analysis

It seems likely that some data may not be available due to voluntary withdrawal of patients, lack of completion of individual data items or general loss to follow-up. Where possible, the reasons for data ‘missingness’ will be ascertained and reported. Although missing data are not expected to be a problem for this study, the nature and pattern of the missingness will be carefully considered—including, in particular, whether the data can be treated as missing completely at random (MCAR). If judged appropriate, missing data will be imputed using the multiple imputation facilities (mice package) available in R (http://www.r-project.org/). The resulting imputed data sets will be analysed and reported, together with appropriate sensitivity analyses. Any imputation methods used for scores and other derived variables, will be carefully considered and justified. Reasons for ineligibility, non-compliance, withdrawal or other protocol violations will be stated and any patterns summarised. More formal analysis, for example, using logistic regression with ‘protocol violation’ as a response, may also be appropriate and aid interpretation.

Standard statistical summaries (eg, medians and ranges or means and variances, dependent on the assumed distribution of the outcome) and graphical plots showing correlations, will be presented for the primary outcome measure and all secondary outcome measures. Baseline data will be summarised to check comparability between treatment arms, and to highlight any characteristic differences between those individuals in the study, those ineligible and those eligible but withholding consent.

The main analysis will investigate differences in the primary outcome measure, the DRI at 6 months after surgery, between the two treatment groups on an intention-to-treat basis. In addition, early functional status will also be assessed and reported at 3 months and later functional status at 12 months. The differences between treatment groups will be assessed using a Student t test, based on a normal approximation for the DRI score at 6 months, and at other occasions. Tests will be two sided and considered to provide evidence for a significant difference if p values are <0.05 (5% significance level). Estimates of treatment effects will be presented with 95% CIs.

As discussed earlier, the stratified randomisation procedure will ensure a balance in recruiting centres between test treatments and as we anticipate that any individual surgeon will operate on no more than 2–3 patients enrolled in the trial, we do not expect surgeon-specific effects to be important in this study. However, in addition to the unadjusted analysis (t tests), we will also undertake regression analyses to adjust for any imbalance between test treatment groups in patient age or gender. The fixed effects analysis (linear regression model) will also be generalised by adding a random effect for recruiting centre to allow for possible heterogeneity in patient outcomes due more generally to the recruiting centre. DRI data will be assumed to be normally distributed during modelling, but subsidiary analyses may also be undertaken after appropriate variance-stabilising transformation. The primary focus will be the comparison of the two treatment groups of patients, and this will be reflected in the analysis, which will be reported together with appropriate diagnostic plots that check the underlying model assumptions. Treatment effects will be presented, with appropriate 95% CIs, for both the unadjusted and adjusted analyses.

Temporal patterns of any complications will be presented graphically and, if appropriate, a time-to-event analysis (Kaplan-Meier survival analysis) will be used to assess the overall risk and risk within individual classes of important complications (eg, infection).

A detailed statistical analysis plan (SAP) will be agreed on with the Data Management Committee (DMC) at the start of the study. Any subsequent amendments to this initial SAP will be clearly stated and justified. Interim analyses will be performed only where directed by the DMC. The routine statistical analysis will mainly be carried out using R (http://www.r-project.org/) and S-PLUS (http://www.insightful.com/). Results from this trial will also be compared with results from other trials.

## Health economic analysis

A prospective economic evaluation, conducted from a National Health Service and personal social services perspective, will be integrated into the trial design.^25^ The economic evaluation will estimate the difference in the cost of resource inputs used by participants in the two arms of the trial, allowing comparisons to be made between the two surgical treatment options (intramedullary nail vs ‘locking’ plate fixation) for patients with a displaced, extra-articular fracture of the distal tibia, and enabling costs and consequences to be compared. The economic assessment method will, as far as possible, adhere to the recommendations of the NICE Reference Case.^25^ Primary research methods will be followed to estimate the costs of the surgical treatment options, including supplementary devices and rehabilitation inputs. Broader resource utilisation will be captured through two principal sources: (1) routine health service data collection systems and (2) patient questionnaires administered at baseline, and 3, 6 and 12 months postrandomisation. Unit costs for health and social care resources will largely be derived from local and national sources and estimated in line with best practice. Primary research using established accounting methods may also be required to estimate unit costs. Costs will be standardised to current prices where possible. Health-related quality of life will be measured at baseline pre-injury status), and 3, 6 and 12 months postoperation, using the EuroQol EQ-5D measure; responses will be used to generate quality-adjusted life-years (QALYs). The EQ-5D is a short questionnaire that is widely used in economic evaluation; utility weights will be taken from the UK General Population tariff for the EQ-5D. We will, in the first instance, use self-reports of the EuroQol EQ-5 D measure. Where these data are not available, we will estimate health utilities at each time point using mapping equations between the DRI score and EQ-5D health outcomes on the basis of existing data sets held by the trial team. Multiple imputation methods will be used to impute missing data and avoid biases associated with complete case analysis. The results of the economic evaluation will be expressed in terms of incremental cost per QALY gained. We shall use non-parametric bootstrap estimation to derive 95% CIs for mean cost differences between the trial groups and to calculate 95% CIs for incremental cost-effectiveness ratios. A series of sensitivity analyses will be undertaken to explore the implications of uncertainty on the incremental cost-effectiveness ratios and to consider the broader issue of the generalisability of the study results. One such sensitivity analysis will involve adopting a societal perspective for the economic evaluation, which will incorporate direct costs to trial participants, informal care provided by family and friends, and productivity losses. In the baseline analysis, and for each sensitivity analysis, cost-effectiveness acceptability curves will be constructed using the net benefits approach. Heterogeneity in the trial population will be explored by formulating net-benefit values for trial participants from the observed costs and effects, and then constructing a regression model with an intervention variable and covariates such as age, contemporaneous injuries and experience of surgeons in trial participating centres. The magnitude and significance of the coefficients on the interactions between the covariates and the intervention variable will provide estimates of cost-effectiveness of the surgical options by participant subgroup. More extensive economic modelling using decision-analytic methods will extend the target population, time horizon and decision context, drawing on best available information from the literature together with stakeholder consultations to supplement the trial data. Parameter uncertainty in the decision-analytic model will be explored using probabilistic sensitivity analysis. Longer term costs and consequences will be discounted to present values using discount rates recommended for health technology appraisal in the UK.

## Discussion

This pragmatic, multicentre trial is due to deliver results in Spring 2017. Results will be disseminated through peer-reviewed publications, including a National Institute for Health Research Health Technology Assessment monograph. Participants of the trial will receive a lay summary of the trial results.
